# Avian reovirus L2 genome segment sequences and predicted structure/function of the encoded RNA-dependent RNA polymerase protein

**DOI:** 10.1186/1743-422X-5-153

**Published:** 2008-12-17

**Authors:** Wanhong Xu, Kevin M Coombs

**Affiliations:** 1 Department of Medical Microbiology and Infectious Diseases, University of Manitoba, Winnipeg, Manitoba R3E 0J9, Canada; 2 Manitoba Centre for Proteomics and Systems Biology, 715 McDermot Avenue, Winnipeg, Manitoba R3E 3P4, Canada

## Abstract

**Background:**

The orthoreoviruses are infectious agents that possess a genome comprised of 10 double-stranded RNA segments encased in two concentric protein capsids. Like virtually all RNA viruses, an RNA-dependent RNA polymerase (RdRp) enzyme is required for viral propagation. RdRp sequences have been determined for the prototype mammalian orthoreoviruses and for several other closely-related reoviruses, including aquareoviruses, but have not yet been reported for any avian orthoreoviruses.

**Results:**

We determined the L2 genome segment nucleotide sequences, which encode the RdRp proteins, of two different avian reoviruses, strains ARV138 and ARV176 in order to define conserved and variable regions within reovirus RdRp proteins and to better delineate structure/function of this important enzyme. The ARV138 L2 genome segment was 3829 base pairs long, whereas the ARV176 L2 segment was 3830 nucleotides long. Both segments were predicted to encode λB RdRp proteins 1259 amino acids in length. Alignments of these newly-determined ARV genome segments, and their corresponding proteins, were performed with all currently available homologous mammalian reovirus (MRV) and aquareovirus (AqRV) genome segment and protein sequences. There was ~55% amino acid identity between ARV λB and MRV λ3 proteins, making the RdRp protein the most highly conserved of currently known orthoreovirus proteins, and there was ~28% identity between ARV λB and homologous MRV and AqRV RdRp proteins. Predictive structure/function mapping of identical and conserved residues within the known MRV λ3 atomic structure indicated most identical amino acids and conservative substitutions were located near and within predicted catalytic domains and lining RdRp channels, whereas non-identical amino acids were generally located on the molecule's surfaces.

**Conclusion:**

The ARV λB and MRV λ3 proteins showed the highest ARV:MRV identity values (~55%) amongst all currently known ARV and MRV proteins. This implies significant evolutionary constraints are placed on dsRNA RdRp molecules, particularly in regions comprising the canonical polymerase motifs and residues thought to interact directly with template and nascent mRNA. This may point the way to improved design of anti-viral agents specifically targeting this enzyme.

## Background

The avian reoviruses (ARVs) are members of the family *Reoviridae*, the only group of dsRNA viruses (out of seven dsRNA virus families) that infect mammals [1,2]. The ARVs are the prototypic members of syncytia-inducing, non-enveloped viruses within the *Orthoreovirus *genus. This genus is divided into 3 subgroups: non-syncytia-inducing mammalian reovirus (MRV; subgroup 1; the prototype of the whole genus), avian reovirus and Nelson Bay virus (subgroup 2), and baboon reovirus (subgroup 3) [3]. In contrast to the MRV, which are rarely associated with human pathology [2,4-6], the ARV are significant pathogens of poultry, and cause a variety of diseases, including infectious enteritis in turkeys [7], viral arthritis/tenosynovitis [8], "pale bird" and runting-stunting syndromes [9], and gastroenteritis, hepatitis, myocarditis, and respiratory illness in chickens [2,8,10].

Like MRV, ARV is a non-enveloped virus with 10 linear double-stranded RNA gene segments surrounded by a double concentric icosahedral capsid shell (inner shell [also called core] and outer shell) of 70–80 nm diameter [11,12]. The ARV genomic segments can be resolved into three size classes based on their electrophoretic mobilities, designated L (large), M (medium), and S (small) [11,12]. In total, the genomic composition includes 3 large segments (Ll, L2, L3), 3 medium sized segments (Ml, M2, M3), and 4 small segments (S1, S2, S3, S4). Nine of the segments are monocistronic and encode a single different protein [11-13] while S1 is tricistronic with partially overlapping open reading frames (ORFs) that encode for three proteins [14,15]. Although ARVs share many features with the prototypic MRVs, several notable differences exist including host range, pathogenicity, hemagglutination properties, and syncytium formation [11,12,16-21].

Genomic coding differences also exist between MRV and ARV. For example, although the ARV and MRV S1 genome segments encode homologous receptor-binding proteins [19,22,23], the ARV S1 genome segment encodes two additional ARV-specific gene products, one of which is responsible for ARV's unusual cell-cell fusion ability [14,15,24], whereas the MRV S1 segment encodes only one additional protein [25]. In addition, available data [12,26] suggest each of the homologous orthoreovirus λ-class proteins are encoded by different ARV and MRV L-class genome segments. Differences in the functional properties of homologous ARV and MRV proteins have also been reported. For example, two non-homologous dsRNA-binding proteins (the ARV σA core protein and the MRV σ3 major outer capsid protein) are predicted to regulate PKR activation [27,28] while the ARV σA core protein displays nucleoside triphosphate phosphohydrolase (NTPase) activity [29], ascribed to the non-homologous MRV μ2 [30] and λ1 [31] core proteins. Based on these early comparative studies, it seems likely that additional analysis of ARV will continue to broaden our understanding of the *Reoviridae *family, possibly leading to the identification of novel features that impact on the distinct biological and pathogenic properties of ARV.

Recent advances have allowed sequence determinations of a growing number of virus isolates. Many ARV and MRV genome segment sequences have been reported. In addition, the complete genomic sequences of three prototype strains of MRV have been completed [32-34]. In contrast, sequence information from ARV isolates is more limited. While the entire complement of S-class genome segments (for example, [14,15,35-39]) and M-class genome segments (for example, [40,41]) have been determined for some ARV clones, and sequence information is available for some ARV L1 and L3 genome segments [42,43], there is, at present, no sequence information for the ARV L2 genome segment. This segment is presumed to encode for the viral RNA-dependent RNA polymerase (RdRp) protein, an essential enzyme for RNA virus replication. Thus, we determined the genomic sequences of the ARV L2 genome segments from two different strains of ARV (ARV138 and ARV176) in order to expand the available ARV sequence database, determine sequences of the ARV RdRp protein, and to delineate conserved structure/function features of this key viral-encoded enzyme.

## Methods

### Cells and viruses

Avian reovirus strain 138 (ARV138) and strain 176 (ARV176) are laboratory stocks. Virus clones were amplified in the continuous quail cell line QM5 in Medium 199 (Gibco) supplemented to contain 7.5% fetal calf serum (Hyclone), 2 mM glutamine, 100 U/ml penicillin, 100 μg/ml streptomycin, and 1 μg/ml amphotericin B, essentially as previously described [44].

### Sequencing the L2 genome segment

Genomic dsRNA was extracted from amplified virus P2 stocks with phenol/chloroform [45]. The extracted dsRNA were resolved in 10% SDS-PAGE and resolved L1, L2, and L3 segments separately excised. Individual segment gel bands were collected into microcentrifuge tubes, macerated, and incubated in 1–2 volumes of diffusion buffer (0.5 M ammonium acetate; 10 mM magnesium acetate; 1 mM EDTA, pH 8.0; 0.1% SDS) at 50°C for 30 minutes. The macerated gel pieces were pelleted by centrifugation at 10,000 × g for 1 min, supernatants were collected and dsRNA precipitated by ethanol. Each pellet was dried and resuspended in ddH_2_O for 3' ligation-based RT-PCR. All primers used for ligation, RT-PCR, and sequencing were synthesized by Invitrogen. An anchor primer, P-5' CTTATTTATTTGCGAGATGGTTATCATTTTAATTATCTCCATG 3'-Bio (5'-end phosphorylated and 3'-end biotin-blocked) was ligated to the 3' end of each genome segment, using T4 RNA ligase according to the manufacturer's instructions (Promega Inc., Madison, USA). Ligated products were precipitated by mixing with 1/2 volume of (30% PEG 8000 in 30 mM MgCl_2_), and centrifuged immediately at 10,000 × g for 30 minutes. The supernatants were removed and pellets were dried and dissolved in ddH_2_O for cDNA synthesis. Full-length cDNA copies of each L2 genome segment were synthesized using a primer (24-mer) complementary to the anchor primer by SuperScript™ II reverse transcriptase according to the manufacturer's instructions (Invitrogen). PCR amplification was performed using cDNA, a forward primer (i.e. primer used for RT), and a reverse primer, 5' ACCGAGGAGAGGgatgaataa 3', designed against highly conserved 3'-end nucleotide sequences of currently known consensus ARV L1 and L3 segment plus strands (shown in lower case) by Expand Long Template PCR System (Roche). PCR products used for DNA sequencing were gel purified using QIAquick^® ^gel extraction kit according to the manufacturer's instructions (Qiagen).

DNA sequencing was performed in both directions by use of an ABI Prism BigDye Terminator v3.1 Cycle Sequencing Ready Reaction Kit (Applied Biosystems) and an Applied Biosystems Genetic Analyzer DNA Model 3100. The first two sequencing reactions were performed with the primers used for PCR amplification. Primers for subsequent reactions were designed from newly obtained sequences to completely sequence each full-length PCR product in both directions. Sequences nearer the ends of each segment were determined from PCR products that were amplified with a primer complementary to the anchor primer and an internal gene-specific primer. Sequences obtained from both directions were assembled and checked for accuracy with SeqMan^® ^(Lasergene^®^, Version 7.1.0; DNASTAR, Inc.).

### Sequence analyses

Sequences were compiled and analyzed using the Lasergene^® ^software suite (Version 7.1.0; DNASTAR, Inc.) Pairwise sequence alignments were performed using the Wilbur-Lipman method [46] for highly divergent nucleotide sequences, the Martinez-NW method [47] for closely related nucleotide sequences, and the Lipman-Pearson method [48] for protein alignments in MegAlign^® ^(Lasergene^®^). Multiple sequence alignments were performed using Clustal-W [49] and T-Coffee [50], and alignment adjustments were manually performed as needed in MegAlign^®^. Amino acid alignment images were adjusted in Adobe Photoshop 7.0 (Adobe^®^). Nucleotide compositions and protein molecular weights were calculated by DNA statistics and protein statistics, respectively, in EditSeq^® ^(Lasergene^®^). Phylogenetic trees were constructed using Neighbor-Joining and tested with 1000 bootstrap replicates in MEGA version 4 [51].

### 3-D structural analyses

Molecular graphics coordinates of the mammalian reovirus (MRV) λ3 crystal structure (PDB # 1MUK; [52]), were manipulated with the UCSF Chimera package from the Resource for Biocomputing, Visualization, and Informatics at the University of California, San Francisco ([53]; supported by NIH P41 RR-01081). Resulting images were imported into Adobe Photoshop and assembled with Adobe Illustrator (Adobe).

## Results

The sequences of genes that encode the RdRp protein have been determined for a number of members of the *Reoviridae *family of viruses (Table 1). However, this information was lacking for members of the avian orthoreovirus subgroup. We determined the sequences of two different strains' ARV L2 genome segments. The L2 genome segments of ARV138 and ARV176 were determined to be 3829 (GeneBank accession no. EU707935) and 3830 (GeneBank accession no. EU707936) nucleotides long, respectively (Table 2). The one-nucleotide length difference is attributed to the 5'-end of the non-translated region of the plus-strand, where ARV138 L2 contains a one-base deletion relative to ARV176 L2. No additional deletions or insertions were found elsewhere in the alignment. The nucleotide identity between ARV138 and ARV176 L2 genome segments is 85% (Table 3). BLAST searches indicated the ARV L2 genome segments were most similar to the mammalian reovirus (MRV) and aquareovirus (AqRV) L1 genome segments, which encode the RNA-dependent RNA polymerase [54,55]. Pairwise sequence comparisons between both of these newly-determined ARV genome segments and all currently available homologous MRV and AqRV L1 genome segments (see Table 1) showed a range of nucleotide and protein identity values. Preliminary comparative studies of all currently available AqRV L-class genome segments indicated that the grass carp reovirus (GCRV) and chum salmon reovirus (CSRV) L genes were the most distantly related amongst the AqRV (data not shown). Thus, although all currently available ARV, MRV, and AqRV L-class genome segments were aligned and compared in subsequent analyses, we limited presentation in subsequent tables and figures to these few most-distant clones for clarity. In addition, preliminary attempts to align the ARV138 and ARV176 L2 genome segments with homologous genes in other *Reoviridae *genera (*ie*. the Fijivirus *Nilaparvata lugens*, the Dinovernavirus *Aedes pseudoscutellaris*, the Coltivirus Eyach virus, the Orbivirus St. Croix River virus, the Seadornavirus Kadipiro virus, the Mimoreovirus *Micromonas pusilla *reovirus, and the currently unclassified virus *Operophtera brumata *reovirus) resulted in much lower identity values and significant gaps (data not shown); thus, these other more-distant genera were not included in subsequent analyses. Pairwise nucleotide sequence comparisons between ARV L2 and homologous MRV genome segments showed identities of ~55%, and pairwise nucleotide sequence comparisons of ARV L2 with AqRV homologues revealed ~48% identity (Table 3).

**Table 1:** Nucleotide sequences used in this study

Strain	GenBank Accession Number
ARV^a^	

138	EU707935

176	EU707936

MRV^b^	

T1L	NC_004271

T2J	NC_004272

T3D	EF494435

T4N	AF368033

BYD1	DQ664184

SC-A	DQ997719

AqRV^c^	

GCRV	AF260512

GCHV	AF284502

GSRV	NC_005167

AGCRV	NC_010585

CSRV	NC_007583

ASRV	EF434978

^a ^ARV, avian reovirus.

^b ^MRV, mammalian reovirus. T1L, type 1Lang; T2J, type 2 Jones; T3D, type 3 Dearing; T4N, type 4 Ndelle.

^c ^AqRV, Aquareovirus. GCRV, Grass carp reovirus; GCHV, Grass carp hemorrhagic virus; GSRV, Golden shiner reovirus; AGCRV, American grass carp reovirus; CSRV, Chum salmon reovirus; ASRV, Atlantic salmon reovirus.

**Table 2:** Genome-segment lengths, non-translated regions, and encoded proteins of ARV138 and ARV176

Genome segment	Base pairs^a^	5' NTR^b^	3' NTR	ORF^c^	Codons^d^	Protein	Molecular weight (kDa)^e^	
		(no. of bases)	(no. of bases)				ARV138	ARV176
L1^f^	3958	20	56	21–3899	1293	λA	142.3	142.2
L2	3829^g^	13^h^	36	14–3790^i^	1259	λB	139.7	139.8
L3^f^	3907	12	37	13–3867	1285	λC	141.9	142.2
M1	2283	12	72	13–2208	732	μA	82.0	82.2
M2	2158	29	98	30–2057	676	μB	73.1	73.3
M3	1996	24	64	25–1929	635	μC	70.9	70.8
S1	1643	24	33	25–318	98	p10	10.3	10.3
				293–730	146	p17	16.9	16.9
				630–1607	326	σC	34.9	34.8
S2	1324	15	58	16–1263	416	σA	46.1	46.1
S3	1202	30	68	31–1131	367	σB	40.9	40.9
S4	1192	23	65	24–1124	367	σNS	40.5	40.6
Total	23492^j^							

^a ^Total nucleotides on each strand.

^b^NTR, non-translated region.

^c^Nucleotide positions indicated for starting and ending codons.

^d^Total number of amino acids in deduced protein.

^e^Molecular weight calculated from deduced protein and rounded to closest 0.1 kDa.

^f^Unpublished.

^g^3830 for ARV176.

^h^14 for ARV176.

^i^15–3791 for ARV176.

^j ^23,493 for ARV176.

**Table 3:** Percent identities of the ARV L2 genome segments and homologous encoded proteins of MRV and Aquareoviruses^a^

Strain	ARV138	ARV176	T1L	T2J	T3D	T4N	GCRV	CSRV
ARV138		98	55	55	55	55	42	41

ARV176	**85**		55	55	55	55	42	41

T1L	**55**	**55**		92	99	97	42	41

T2J	**55**	**55**	**75**		92	91	42	40

T3D	**55**	**55**	**96**	**76**		98	42	41

T4N	**56**	**56**	**89**	**75**	**90**		42	41

GCRV	**49**	**49**	**48**	**47**	**48**	**47**		58

CSRV	**47**	**47**	**47**	**46**	**47**	**47**	**59**	

^a ^Percent amino acid identities indicated in upper triangle; percent nucleotide identities are in lower triangle, in bold.

**Figure 1:**
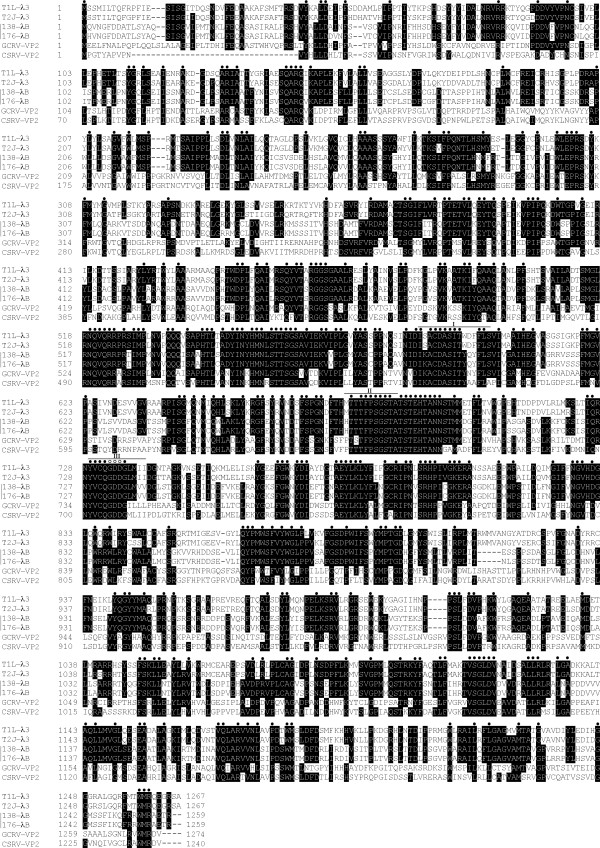
**Alignment of the deduced ARV138 and ARV176 λB amino acid sequences**. All 14 currently available homologous ARV λB, MRV λ3, and AqRV VP2 proteins (determined for each clone shown in Table 1) were aligned, both by T-Coffee [50] (data not shown) and by Clustal-W [49], with only minor differences in the alignments created by different gap penalties (data not shown). Only the two most-distant ARV, MRV, and AqRV sequences (see text for details) are shown for clarity. Clones are: MRV – T1L (GenBank No. NC_004271) and T2J (GenBank No. NC_004272); ARV – ARV138 (GenBank No. EU707935) and ARV176 (GenBank No. EU707936); AqRV – Grass Carp reovirus (GCRV) (GenBank No. AF260512) and Chum Salmon reovirus (CSRV) (GenBank No. NC_007583). Amino acid residues that are identical in at least four of the sequences are indicated by black background shading. The single letter amino acid code is used. Previously identified polymerase domains (labeled I – III) [56] are indicated with solid horizontal lines above the sequences. Amino acid residues that are completely conserved in all 14 sequences are indicated by closed circles, and the GDD motif found in all polymerases is indicated by open circles, shown above the sequences.

**Figure 2:**
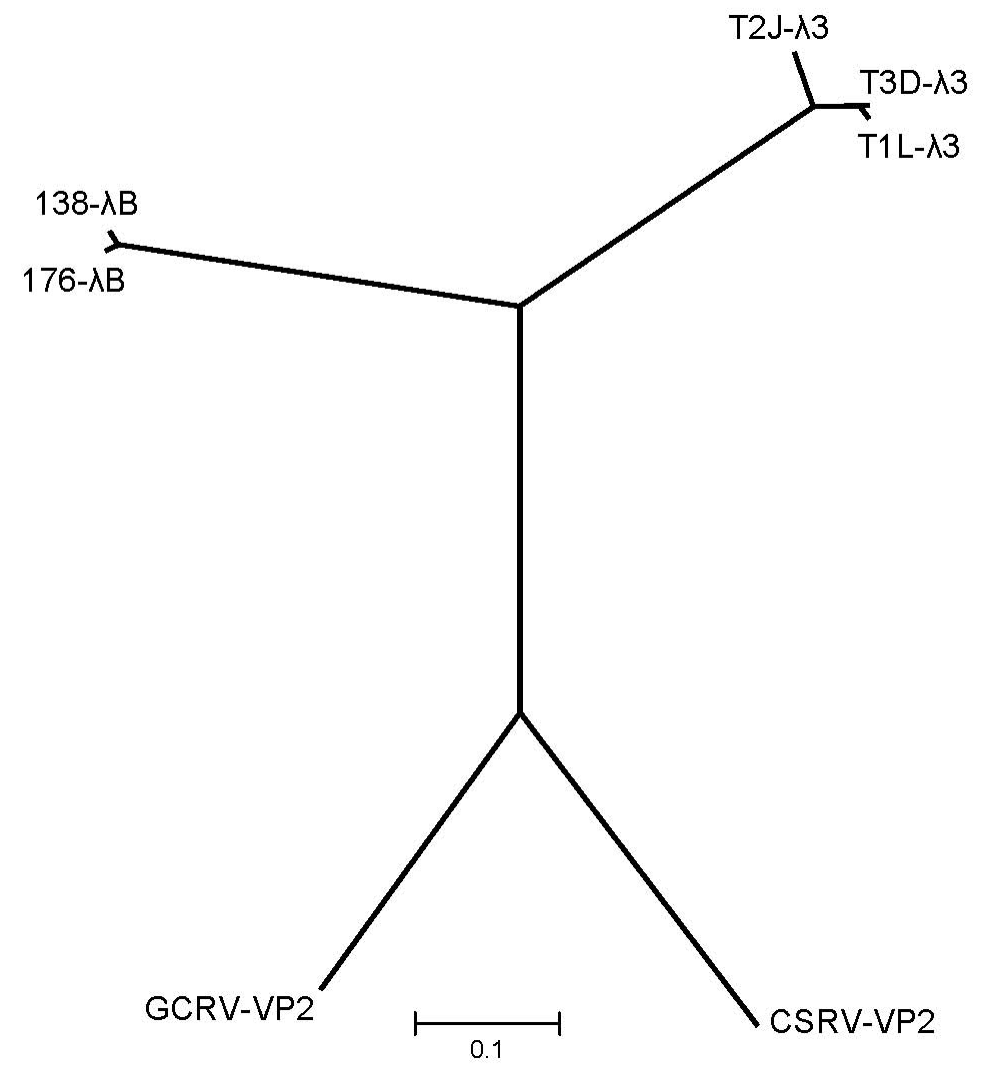
**Phylogenetic tree analyses of the prototype ARV L2 genome segments and homologous genes in other reoviruses**. Abbreviations are as defined in the legend to Fig. 1. Lines are proportional in length to nucleotide substitution. Alignments were performed by Neighbor-Joining and tested with 1000 bootstrap replicates in MEGA version 4 [51].

**Figure 3:**
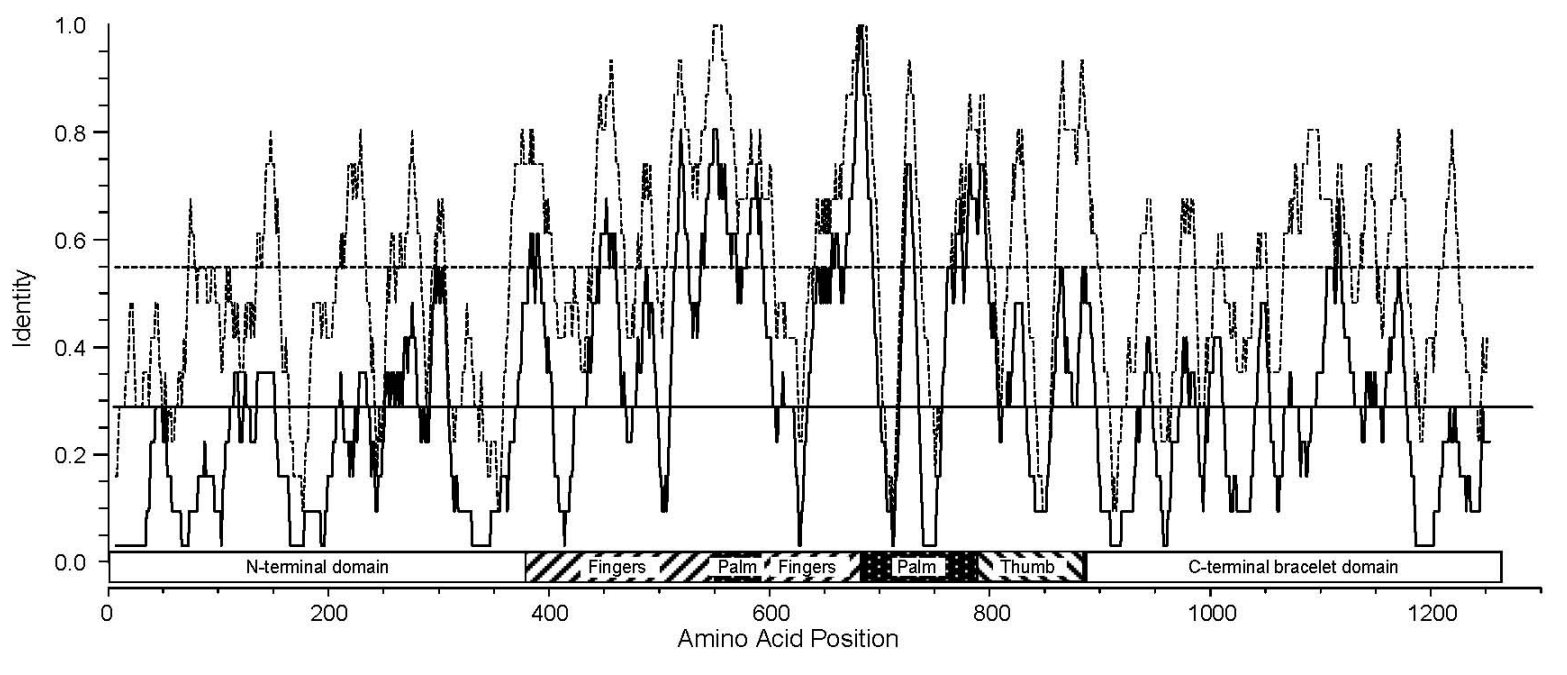
**Window-averaged scores for sequence identity among the ARV λB, AqRV VP2, and MRV λ3 RNA-dependent RNA polymerase proteins**. To provide consistent weighting to the averaged scores, only the two most-distant clones from each of the three groups (ARV: ARV138 and ARV176; AqRV: GCRV and CSRV; MRV: T1L and T2J – see text for details) were used. Identity scores averaged over running windows of 15 amino acids and centered at consecutive amino acid positions are shown for ARV:MRV comparisons (dashed lines) and ARV:MRV:AqRV comparisons (solid line). The global identity scores for each of the compared sequence sets are indicated by the horizontal lines. Previously-identified enzymatic motifs are indicated with boxes below the plots.

The predicted open reading frames for both ARV L2 segments were determined to be nucleotides 14–3790 for ARV138 L2 and 15–3791 for ARV176 L2, resulting in deduced λB proteins of 1259 residues (Table 2). The calculated molecular weights for ARV138 λB and ARV176 λB are ~140 kDa each (Table 2). The amino acid identity between the two ARV λB proteins is 97.5%, with no insertions or deletions relative to one another. ARV protein λB is the only ARV protein whose sequence has not been reported previously. Thus, completion of the L2 sequence in this study has allowed us to assign its function at the sequence level. Amino acid alignments of ARV λB, MRV λ3, and AqRV VP2 proteins revealed several regions of high amino acid identity (Fig. 1), many of which correspond to previously identified polymerase domains [56]. A large number of amino acids were completely conserved across all 14 currently known ARV, MRV, and AqRV RdRp protein sequences (Fig. 1, closed circles). Amino acid identities between ARV λB and homologous MRV λ3 or AqRV VP2 are ~55% and ~42%, respectively (Table 3), suggesting the ARV and MRV are more closely related to each other than either are to AqRV (also seen in phylogenetic analysis – Fig. 2), reflecting that ARV and MRV belong to different species in the *Orthoreovirus *genus [36] whereas AqRV are members of the different *Aquareovirus *genus in the *Reoviridae *family. Window-averaged analysis of ARV λB and MRV λ3 protein identities (Fig. 3, dashed lines) revealed several regions of high amino acid identity. The highest identity scores, with window-averaged identity values > 90%, were located within canonical polymerase regions, including "fingers" domains (MRV residues 452 – 467 and 514 – 530) "fingers"/"palm" interface domains (MRV residues 542 – 571 and 673 – 699), "palm" domains (MRV residues 725 – 738, which includes the GDD motif, which is common to all viral RNA-dependent RNA polymerases [57-59]), "thumb" domains (MRV residues 864 – 878), and an "undefined" domain (MRV residues 881 – 896). Addition of the AqRV VP2 protein to the above analyses provided additional information about potentially important conserved domains. Clustal-W (Fig. 1) and T-Coffee (data not shown) alignments identified 359 amino acid residues that were identical in the 6 aligned sequences (overall average identity = 28.3% Fig. 3, horizontal solid line]). There were numerous window-averaged regions of very low conservation, with most attributed to AqRV regions that were poorly conserved compared to corresponding ARV/MRV regions, a feature also noted in MRV:AqRV comparisons [60]. Three regions showed higher-than-average conservation in the ARV:MRV:AqRV alignments, with window-averaged identity values > 75%, suggesting these polymerase regions (ARV residues G_516_LRNQVQRRPRTIMP_530_, H_542_TLS/CADYINYHMNLSTTSGSAV_563_, and T_677_TTFPSGSTATSTEHTANNSTM_698_, that correspond to MRV residues G_516_LRNQVQRRPRSIMP_530_, H_542_TLTADYINYHMNLSTTSGSAV_563_, and T_677_TTFPSGSTATSTEHTANNSTM_698_, respectively) contain important structural/functional domains. The GDD motif was located within a region of slightly lower window-averaged scores (~60%), but in a sequence (in ARV) I_724_QxxYVCQGDDG_735 _that, apart from the residues at positions 726 and 727, were completely conserved in all 14 currently-available ARV, MRV, and AqRV RdRp sequences. In addition to the 359 identical residues found in all 6 sequences discussed above, blossum50 weighting alignments indicated that an additional 206 positions contained either identical amino acid residues or conservative substitutions in at least 4 of the 6 aligned sequences.

## Discussion

**Figure 4:**
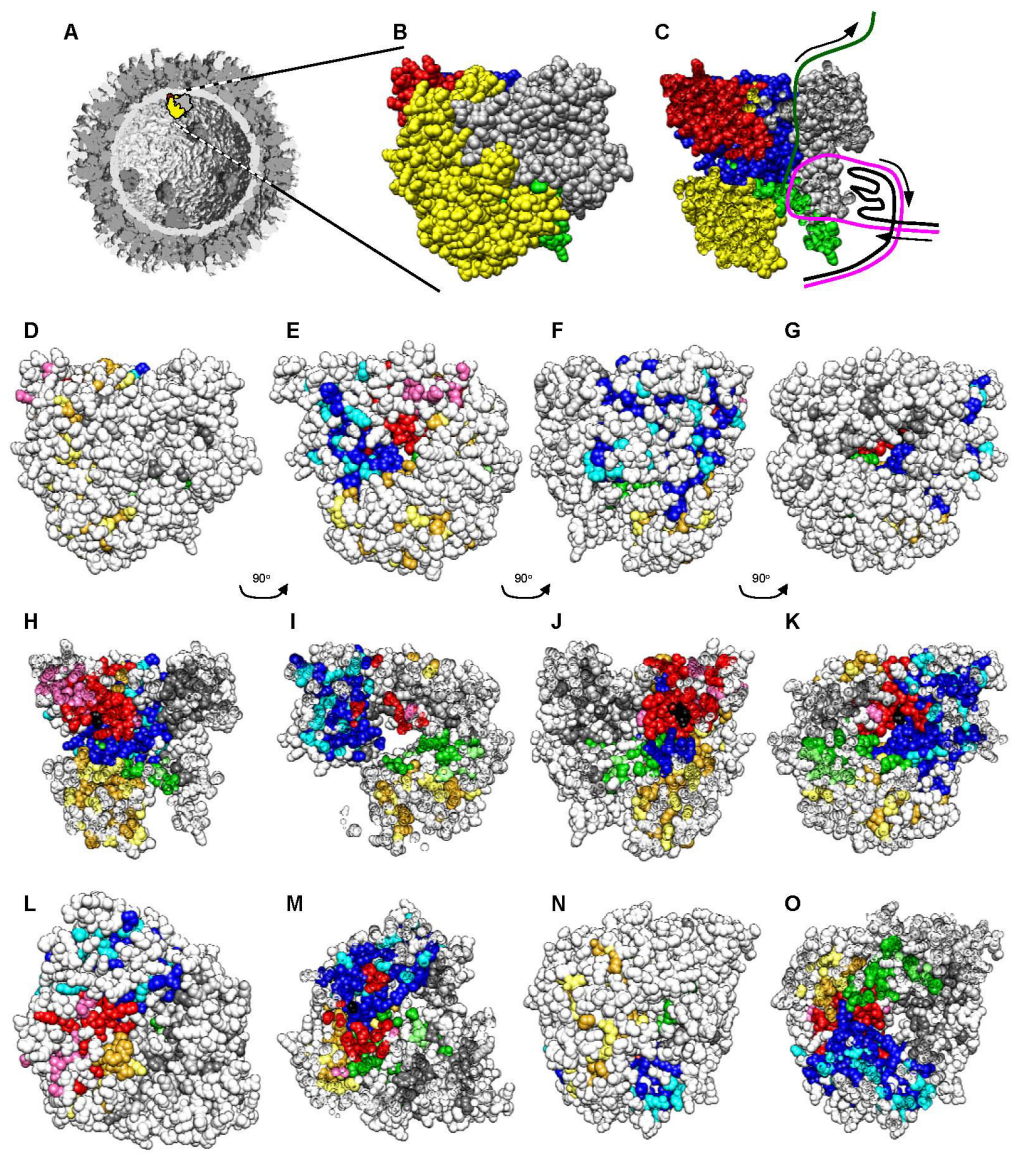
**Localization of conserved, non-conserved, and identical amino acids in ARV, MRV, and AqRV RdRp proteins**. The MRV λ3 crystal structure (PDB # 1MUK [52]) was manipulated with Chimera [53]. **A**, Low-resolution, cutaway model of the reovirus core structure (modified from [26] with permission). **B**, Blow-up of indicated λ3 molecule in 'A', and **C**, cut-away of "B" with presumptive paths of genomic (+) RNA (black line), template genomic (-) RNA (magenta line) and nascent mRNA (dark green line) shown (adapted from and as described in [62]); Specific motifs in 'B' – 'O' are color-coded, with N-terminal region in yellow, C-terminal "bracelet" in grey, and canonical polymerase "fingers", "palm", and "thumb" depicted in blue, red, and green, respectively. **D**, Same as 'B', but in "D" – "O", amino acids that are identical in all 6 ARV, MRV, and AqRV sequences (see Fig. 1) are shown in darker versions of each motif color (goldenrod, dim grey, blue, red, and green, respectively), amino acids that represent conservative substitutions (as determined by Blossum50 matrix) are shown in lighter versions of each motif color (yellow, medium grey, cyan, hotpink, and light green, respectively), and non-conserved amino acids are shown in white. The canonical GDD motif is depicted in black. **D - G**, represent successive 90° rotations counter-clockwise around vertical axis, of entire RdRp protein, to correspond to front (as depicted in "A"), left side, back, and right side. **H - K**, represent same views as "D - G", respectively, but with the front approximate half of each view removed. **L **and **N**, represent top and bottom view, respectively, of RdRp molecule. **M**, represents top view, after upper approximately 40% of view removed, and **O**, represents bottom view, after lower approximately half of view removed. The top surface depicted in "L" is believed to interact with the λ-class core shell protein.

The atomic structure of few ARV proteins have been reported [61], and such high-resolution structures are not known for any λ-class ARV proteins. By contrast, atomic structures are known for most MRV proteins, including the RdRp [52]. Comparative sequence analyses described in this report have indicated that ARV and MRV RdRp proteins share ~55% amino acid identity, ARV and AqRV RdRp proteins share ~42% identity, and that only 359 (~28%) amino acids are completely conserved (identical) when ARV138, ARV176, MRV T1L, MRV T2J, AqRV CSRV, and AqRV GCRV are aligned (Fig. 1). Thus, to gain structure/function information about this key viral-encoded enzyme, ARV, MRV, and AqRV identical amino acids, conservative substitutions, and non-conservative substitutions were modeled in the MRV λ3 crystal structure (Fig. 4). This comparative analysis indicated that most non-conserved amino acids were located on the surfaces of the protein exposed to the core interior and in the N-terminal and C-terminal bracelet domains, whereas most identical amino acids and conservative substitutions were located within canonical fingers, palm, and thumb polymerase motifs, particularly those lining channels used by template and nascent RNA during transcription (Fig. 4). Similar observations had been reported from MRV:AqRV comparisons [60] and our results support and extend these earlier observations. As was previously reported from MRV:AqRV comparisons [60], conserved residues surround the GDD motif and additional residues shown to be important for a variety of polymerase functions are also conserved, including Arg_522_, Arg_523_, Arg_525_, Ala_587 _(which are needed to properly position the incoming NTP triphosphate), Ile_527 _and Pro_529 _(needed to help position template nucleosides), Thr_557_, Ser_558_, Gly_559_, Ser_560_, and Val_562 _(portions of a loop that maintains priming NTP), and Asp_589_, Ser_681_, and Gln_731 _(specifies ribonucleotides). Each of these residues is located one amino acid more N-terminal in the MRV sequence (ie. ARV Arg_522 _= MRV Arg_523 _and all (as well as numerous others) are completely conserved in all 14 currently available ARV, MRV, and AqRV RdRp sequences (Fig. 1, indicated by closed circles). In addition, our comparative analyses indicated many identical amino acids and conservative substitutions were located on the surface of the protein that is predicted to interact with the core shell [62]. This might imply that conserved domains are needed to help tether the RdRp to the underside of the core shell. This hypothesis could be tested by extending such ARV:MRV:AqRV sequence comparisons to the other core proteins.

In conclusion, we report the first sequence analysis of the avian reovirus RdRp gene and protein. The ARV λB and MRV λ3 proteins showed the highest ARV:MRV identity values (~55%) amongst currently known ARV and MRV proteins, suggesting significant evolutionary constraints are placed on dsRNA RdRp molecules, particularly in regions comprising the canonical polymerase motifs and residues thought to interact directly with template and nascent mRNA.

## Competing interests

The authors declare that they have no competing interests.

## Authors' contributions

WX performed the experiments and analyses and WX and KC wrote the manuscript.
